# Correction: Yu et al. Nitrogen Doped Porous Biochar/β-CD-MOFs Heterostructures: Bi-Functional Material for Highly Sensitive Electrochemical Detection and Removal of Acetaminophen. *Molecules* 2023, *28*, 2437

**DOI:** 10.3390/molecules29214979

**Published:** 2024-10-22

**Authors:** Qi Yu, Jin Zou, Chenxiao Yu, Guanwei Peng, Guorong Fan, Linyu Wang, Shangxing Chen, Limin Lu, Zongde Wang

**Affiliations:** 1 East China Woody Fragrance and Flavor Engineering Research Center of NF&GA, College of Forestry, Jiangxi Agricultural University, Nanchang 330045, China; 2 Key Laboratory of Crop Physiology, Ecology and Genetic Breeding, Ministry of Education, Key Laboratory of Chemical Utilization of Plant Resources of Nanchang, College of Chemistry and Materials, Jiangxi Agricultural University, Nanchang 330045, China

Following publication, concerns were raised regarding the peer-review process related to the publication of this article [1]. Adhering to our standard procedure, the Editorial Board conducted an investigation which determined that, while the peer-review process does comply with MDPI’s Editorial Process policy (https://www.mdpi.com/editorial_process), the contribution of one of the three reviewers does not comply with MDPI’s Guideline for Reviewers (https://www.mdpi.com/reviewers#_bookmark11) nor the expectations of the Editorial Board. As a result, the Editorial Board has decided to remove the contribution of one of the reviewers from the open peer-review record (https://www.mdpi.com/1420-3049/28/6/2437/review_report) and, following discussion with the authors, have removed the citations (references [4,8] in the original publication) recommended by this reviewer from this publication [1]. The Editorial Board has confirmed that the publication of this paper in its modified form is in line with their expectations and MDPI’s Editorial Process policy. The authors state that the scientific conclusions are unaffected. The publication and associated webpage have been updated accordingly.
