# Supplementary material for: Nitrogen Doped Porous Biochar/β-CD-MOFs Heterostructures: Bi-Functional Material for Highly Sensitive Electrochemical Detection and Removal of Acetaminophen
Source: Molecules. 2023 Mar 7;28(6):2437. doi: 10.3390/molecules28062437 (PMC10054116; doi:10.3390/molecules28062437)
Supplement: Supplementary file 1 [file molecules-28-02437-s001.zip › molecules-2196222-supplementary.pdf]

Supplementary Materials

Nitrogen Doped Porous Biochar/β-CD-MOFs Heterostructures: Bi-functional Material for Highly Sensitive Electrochemical Detection and Removal of Acetaminophen

Qi Yu [1,2], Jin Zou [1], Chenxiao Yu [2], Guanwei Peng [2], Guorong Fan [1], Linyu Wang [2,*], Shangxing Chen [1], Limin Lu [2,*] and Zongde Wang [1,*]

[1] East China Woody Fragrance and Flavor Engineering Research Center of NF&GA, College of Forestry, Jiangxi Agricultural University, Nanchang 330045, China; qiyujxau@163.com (Q.Y.); 15797710413@163.com (J.Z.); fgr008@126.com (G.F.); csxing@jxau.edu.cn (S.C.)

[2] Key Laboratory of Crop Physiology, Ecology and Genetic Breeding, Ministry of Education, Key Laboratory of Chemical Utilization of Plant Resources of Nanchang, College of of chemistry and materials, Jiangxi Agricultural University, Nanchang 330045, China; xxynllrbkq111@163.com (C.Y.); pengguanwei163@163.com (G.P.)

* Correspondence: linyuwang@jxau.edu.cn (L.W.), lulimin816@126.com (L.L.), zongdewang@163.com (Z.W.)

1 Reagents

AC, melamine, β-CD, KOH, chitosan, HCl, NaH_2PO_4 and Na_2HPO_4 were purchased from Aladdin (Shanghai, China). Soulangeana sepals were obtained from the botanical garden of Jiangxi Agricultural University (Nanchang, Jiangxi). Phosphate buffered solutions (PBS, 0.1 M) with different pH were prepared with 0.1 M Na_2HPO_4 and 0.1 M NaH_2PO_4. Ultrapure water was used in the experimental.

2 Apparatuses

Scanning electron microscopy (SEM, Hitachi S-4800, Japan) was used to observe the morphology and microstructure of the N-SC/β-CD-MOFs nanocomposites. X-ray diffraction (XRD) patterns of different materials were recorded by the X-ray powder diffractometer (Lab XRD-6100). The surface chemical composition of N-SC/β-CD-MOFs were evaluated by X-ray photoelectron spectroscopy (XPS) on the ESCALAB

250Xi instrument (ThermoFischer, USA). In the adsorption experiment, the concentration of acetaminophen was determined by UV-Vis spectrophotometer (UV-1800, Shimadzu, Japan). All electrochemical experiments were performed on a CHI760E workstation (Shanghai, China) with a conventional three-electrode system, including a saturated calomel electrode as the reference electrode, a platinum sheet electrode as the counter electrode, and a glassy carbon electrode (GCE, 3 mm) as the working electrode.

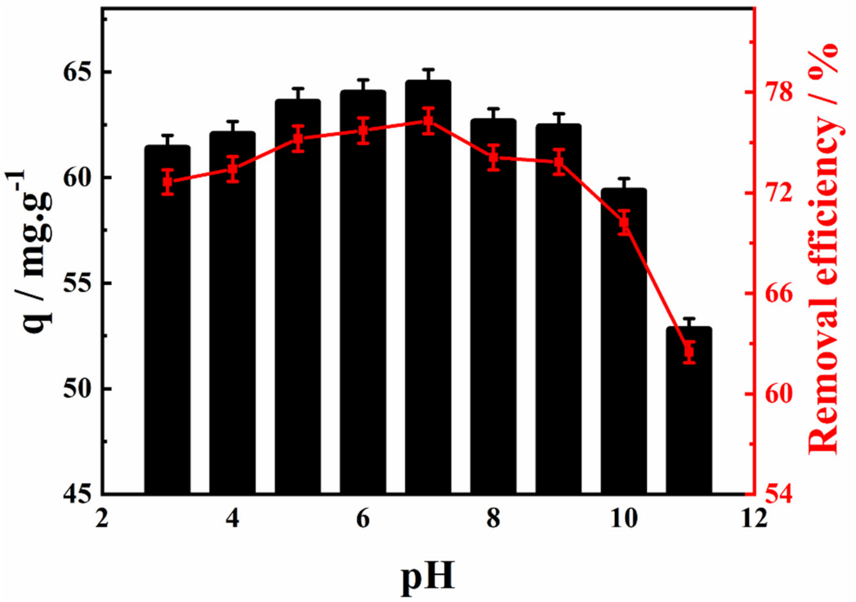

Figure S1. Effect of pH for the adsorption of AC on N-SC/β-CD-MOFs.

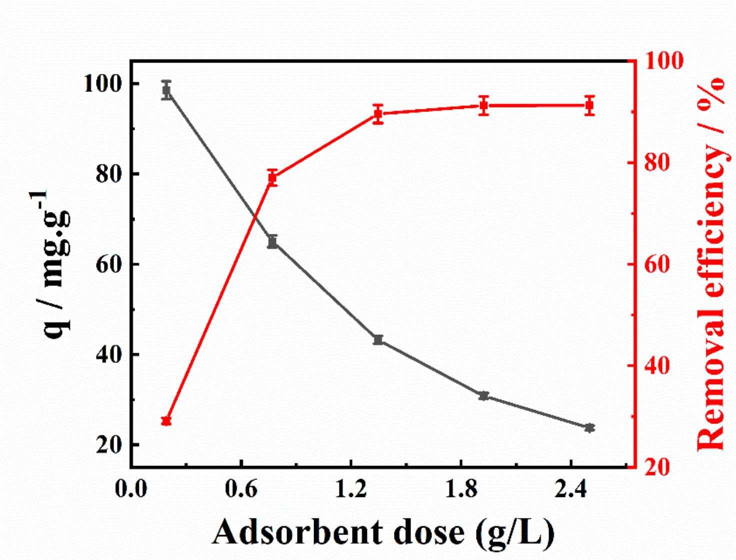

Figure S2. Effect of N-SC/β-CD-MOFs dose towards AC adsorption.

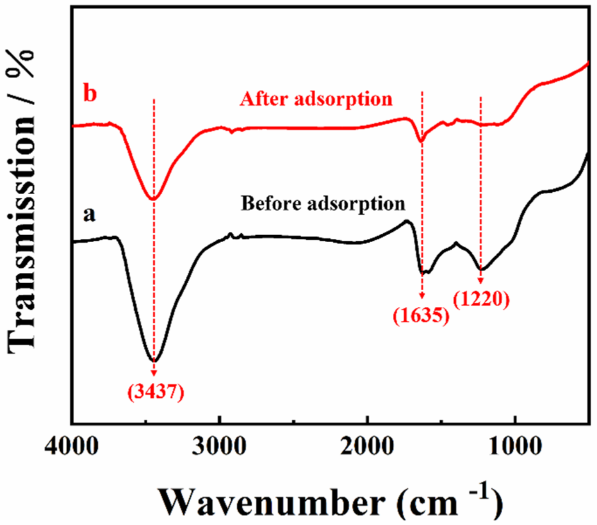

Figure S3. FTIR spectrum of N-SC/β-CD-MOFs before and after adsorption of AC.

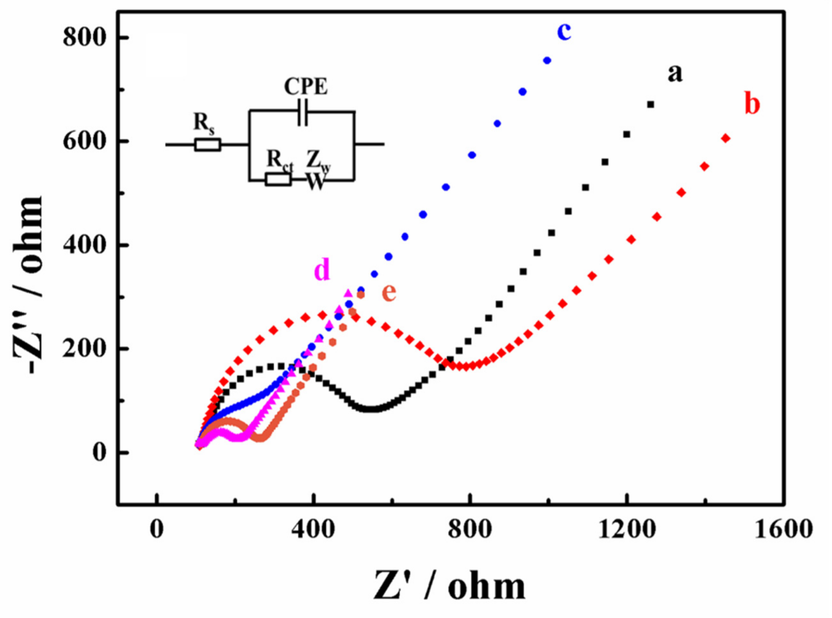

Figure S4. Nyquist plot of bare GCE (curve a), β-CD-MOFs/GCE (curve b), SC/GCE (curve c), N-SC/GCE (curve d) and N-SC/β-CD-MOFs/GCE (curve e) in 0.1 M KCl containing 5.0 mM $[Fe(CN)6]^{3-/4-}$.

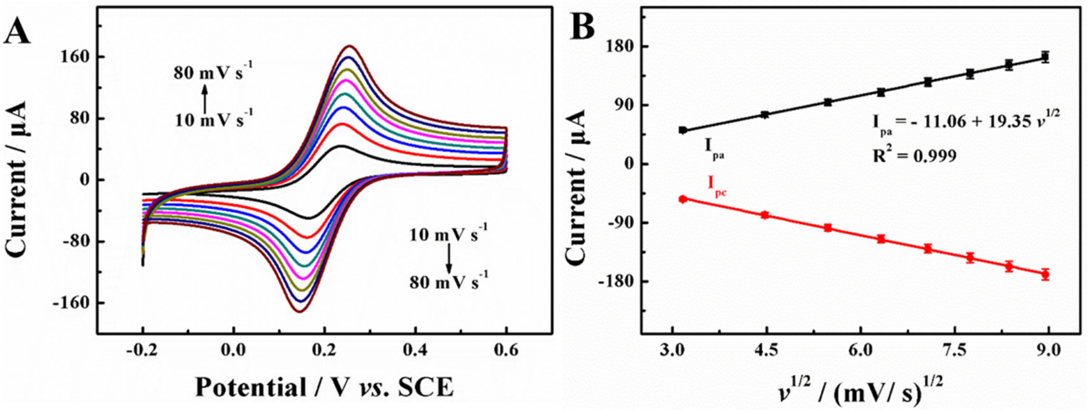

Figure S5. (A) CV curves of N-SC/β-CD-MOFs/GCE in 0.1 M KCl containing 5 mM $[Fe(CN)_6]^{3-/4-}$ under different scan rates; (B) The fitting curve of the peaks current *vs*. the square root of scan rates.

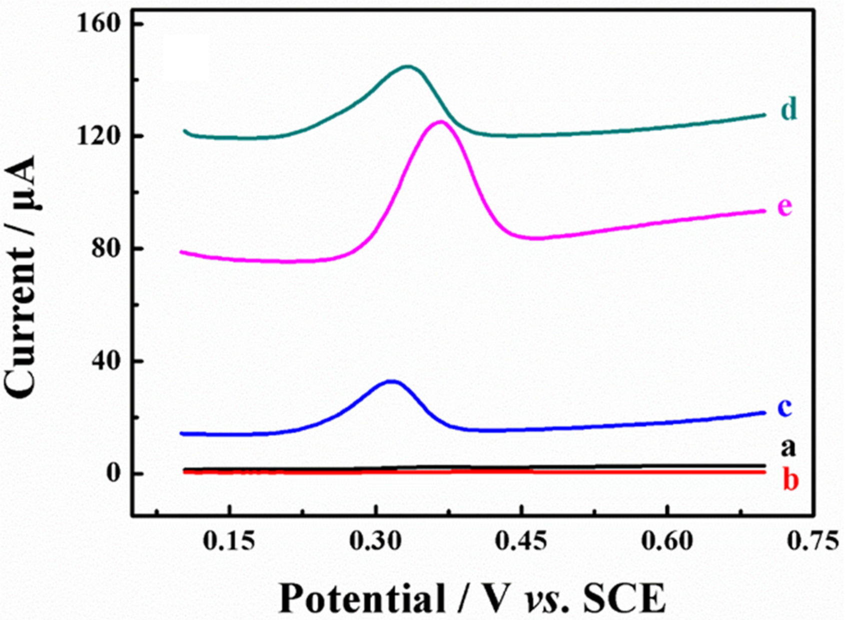

Figure S6. DPVs behaviors of 20.0 μM AC in 0.1 M PBS (pH=7.0) on bare GCE (a), β-CD-MOFs/GCE (b), SC/GCE (c), N-SC/GCE (d) and N-SC/β-CD-MOFs/GCE (e). (pulse width is 0.05 s, amplitude is 50 mV and accumulation time is 180 s).

Table S1. Comparison of adsorption capacities of different adsorbents for AC.

Adsorbent	Adsorption equilibrium time (min)	Adsorption capacity (mg/g)	Isotherm type	References
Pyrolyzed pulp mill sludge	80	19.74	Langmuir	[1]
Oak fruits	100	45.45	Langmuir	[2]
Carbon nanotubes	60	1.99	Langmuir	[3]
Natural Jordanian zeolite	80	55.60	Langmuir	[4]
Seed husks (M.oleifera)	450	17.48	Langmuir	[5]
Polymeric resin	1000	6.90	Langmuir-Freundlich	[6]
Activated Coconut shell	1800	38.20	Experimental	[7]
N-SC/β-CD-MOFs	30	66.43	Langmuir	This work

Table S2. Comparison of the detection performance of AC by N-SC/β-CD-MOFs/GCE and other electrochemical sensors.

Modified electrodes	Method	Linear range (μM)	LOD (nM)	Reference
Au@Fe_3O_4@rGO/GCE	DPV	0.01-0.28	5.0	[8]
P-NC [a]/GCE	DPV	3-110	500	[9]
Pd-MWCNTs [b]/GCE	DPV	0.5-100	130	[10]
PB-MWCNTs-COOH/ZIF-67/GCE	DPV	0.01-70.0	3.3	[11]
P-RGO [c]/GCE	DPV	1.5-120	360	[12]
N-SC/β-CD-MOFs/GCE	DPV	0.001-30.0	0.3	This work

a P-NC: nitrogen-rich porous carbon;

b MWCNTs: The negatively charged carboxylated multi-walled carbon nanotubes;

c P-RGO: Phosphorus-doped graphene.

Table S3. Detection of AC in lake water samples with N-SC/β-CD-MOFs/GCE (n=5).

Samples	Added (μM)	Found (μM)	RSD (%)	Recovery (%)
1	0	-	-	-
2	1.0	0.95 ± 0.15	3.25	95.0
3	3.0	2.87 ± 0.18	2.82	95.7
4	5.0	5.21 ± 0.14	2.27	104.2
5	10.0	9.93 ± 0.23	2.98	99.3

References

1 Gil, A.; Taoufik, N.; García, A. M.; Korili, S. A. Comparative removal of emerging contaminants from aqueous solution by adsorption on an activated carbon. *Environ Technol.* **2019**, 40, 3017-3030.

2 Rakić, V.; Rac, V.; Krmar, M.; Otman, O.; Auroux, A. The adsorption of pharmaceutically active compounds from aqueous solutions onto activated carbons. *J. Hazard. Mater.* **2015**, 282, 141-149.

3 Wang, Y.; Wei, X.; Zhang, R.; Wu, Y.; Farid, M. U.; Huang, H. Comparison of chemical, ultrasonic and thermal regeneration of carbon nanotubes for acetaminophen, ibuprofen, and triclosan adsorption. *RSC Adv.* **2017**, 7, 52719-52728.

4 Al-rimawi, F.; Daana, M.; Khamis, M.; Karaman, R.; Khoury, H.; Qurie, M. Removal of Selected Pharmaceuticals from Aqueous Solutions Using Natural Jordanian Zeolite. *Arab. J. Sci. Eng.* **2019**, 44, 209-215.

5 Quesada, H. B.; Cusioli, L. F.; O Bezerra, C.; Baptista, A. T.; Nishi, L.; Gomes, R. G.; Bergamasco, R. Acetaminophen adsorption using a low-cost adsorbent prepared from modified residues of Moringa oleifera Lam. seed husks. *J. Chem. Technol. Biotechnol.* **2019**, 94, 3147-3157.

6 Coimbra, R.; Escapa, C.; Otero, M. Adsorption Separation of Analgesic Pharmaceuticals from Ultrapure and Waste Water: Batch Studies Using a Polymeric Resin and an Activated Carbon. *Polymers.* **2018**, 10, 958.

7 Yanyan, L.; Kurniawan, T. A.; Zhu, M.; Ouyang, T.; Avtar, R.; Dzarfan Othman, M. H.; Mohammad, B. T.; Albadarin, A. B. Removal of acetaminophen from synthetic wastewater in a fixed-bed column adsorption using low-cost coconut shell waste pretreated with NaOH, HNO_3, ozone, and/or chitosan. *J. Environ. Manage.* **2018,** 226, 365-376.

8 Venkatachalam, R.; Annadurai, T.; Nesakumar, N.; Vembu, S. Fortified electrochemical activity of Au@Fe_3O_4@rGO decorated GCE for sensing of acetaminophen. *Mater. Today Commun.* **2021**, 27, 102236.

9 Liang, W.; Liu, L.; Li, Y.; Ren, H.; Zhu, T.; Xu, Y.; Ye, B. Nitrogen-rich porous carbon modified electrochemical sensor for the detection of acetaminophen. *J. Electroanal. Chem.* **2019**, 855, 113496.

10 Gao, F.; Zou, J.; Zhong, W.; Tu, X.; Huang, X.; Yu, Y.; Wang, X.; Lu, L.; Bai, L. Prussian blue-carboxylated MWCNTs/ZIF-67 composite: a new electrochemical sensing platform for paracetamol detection with high sensitivity. *Nanotechnology.* **2020**, 32, 085501.

11 Wu, Y.; Wu, Y.; Lv, X.; Lei, W.; Ding, Y.; Chen, C.; Lv, J.; Feng, S.; Chen, S.; Hao, Q. A sensitive sensing platform for acetaminophen based on palladium and multi-walled carbon nanotube composites and electrochemical detection mechanism. *Mater. Chem. Phys.* **2020**, 239, 121977.

12 Zhang, X.; Wang, K.; Zhang, L.; Zhang, Y.; Shen, L. Phosphorus-doped graphene-based electrochemical sensor for sensitive detection of acetaminophen. *Anal. Chim. Acta.* **2018**, 1036, 26-32.
